# Concomitant attenuation of HMGCR expression and activity enhances the growth inhibitory effect of atorvastatin on TGF-β-treated epithelial cancer cells

**DOI:** 10.1038/s41598-021-91928-3

**Published:** 2021-06-17

**Authors:** Katsuhiko Warita, Takuro Ishikawa, Akihiro Sugiura, Jiro Tashiro, Hiroaki Shimakura, Yoshinao Z. Hosaka, Ken-ichi Ohta, Tomoko Warita, Zoltán N. Oltvai

**Affiliations:** 1 Department of Veterinary Anatomy, School of Veterinary Medicine, Tottori University, 4-101 Koyama Minami, Tottori, Tottori 680-8553 Japan; 2 Department of Anatomy and Neurobiology, Faculty of Medicine, Kagawa University, 1750‐1 Miki-cho, Kita-gun, Kagawa 761-0793 Japan; 3 Department of Bioscience, School of Science and Technology, Kwansei Gakuin University, 2‐1 Gakuen, Sanda, Hyogo 669‐1337 Japan; 4 Department of Pathology and Laboratory Medicine, University of Rochester, 601 Elmwood Ave, Rochester, NY 14642 USA

**Keywords:** Cancer, Oncology

## Abstract

Epithelial-mesenchymal transition (EMT) in primary tumor cells is a key prerequisite for metastasis initiation. Statins, cholesterol-lowering drugs, can delay metastasis formation in vivo and attenuate the growth and proliferation of tumor cells in vitro. The latter effect is stronger in tumor cells with a mesenchymal-like phenotype than in those with an epithelial one. However, the effect of statins on epithelial cancer cells treated with EMT-inducing growth factors such as transforming growth factor-β (TGF-β) remains unclear. Here, we examined the effect of atorvastatin on two epithelial cancer cell lines following TGF-β treatment. Atorvastatin-induced growth inhibition was stronger in TGF-β-treated cells than in cells not thusly treated. Moreover, treatment of cells with atorvastatin prior to TGF-β treatment enhanced this effect, which was further potentiated by the simultaneous reduction in the expression of the statin target enzyme, 3-hydroxy-3-methylglutaryl coenzyme A reductase (HMGCR). Dual pharmacological targeting of HMGCR can thus strongly inhibit the growth and proliferation of epithelial cancer cells treated with TGF-β and may also improve statin therapy-mediated attenuation of metastasis formation in vivo.

## Introduction

Cholesterol-lowering drugs, statins, are the most frequently prescribed agents for the treatment of hyperlipidemia, thus alleviating cardio- and cerebrovascular morbidity and mortality^1^. Mechanistically, statins inhibit the activity of 3-hydroxy-3-methylglutaryl coenzyme A (HMG-CoA) reductase (HMGCR), the rate-limiting enzyme of the mevalonate pathway^2^. Statins also exert anticancer effects, partly by inhibiting small G-protein prenylation and proinflammatory cytokine secretion^3^. Statins are preferentially effective against mesenchymal-like cancer cells that express the mesenchymal marker vimentin but do not express E-cadherin on the cell membrane. In contrast, epithelial cancer cells that express E-cadherin on their cell surface display relative resistance to statin treatment^4–6^.

Epithelial-mesenchymal transition (EMT) is a process wherein epithelial cells lose their polarity and cell–cell adhesion and gain migratory and invasive properties. Moreover, EMT can induce drug resistance, immunosuppression and apoptosis escape^7,8^. Cells undergoing EMT also acquire stem cell-like functions and can transform into cancer stem cells^9,10^.

Transforming growth factor-β (TGF-β) is a multifunctional cytokine that can induce EMT by stimulating the transcription of target genes, mainly via the Smad2/3 pathway^11–13^. Several cell types secrete TGF-β in the tumor microenvironment, including cancer cells and surrounding normal cells such as fibroblasts, endothelial cells, mesenchymal cells, and adipocytes^14^. Additionally, elevated blood levels of TGF-β in cancer patients are associated with poor prognosis^15,16^.

Metastases lead to approximately 90% of cancer-related deaths^17^, and EMT is considered an important first step in metastasis initiation. We previously reported that mesenchymal-like tumor cells displayed increased sensitivity to atorvastatin-induced growth delay^4,18^. Atorvastatin treatment also reduced metastasis formation in the lung and liver in two independent mouse models of spontaneous breast cancer metastasis without affecting growth in their respective primary tumors^19^. These data collectively suggest that cancer cells that initiated, undergo or have completed EMT are more susceptible to statins’ inhibitory effects than those that do or have not, both in vitro and in vivo.

In this study, we examined whether TGF-β treatment of epithelial-like cancer cells and the concomitant downregulation of *HMGCR* expression counteracted the cells’ resistance to atorvastatin. We also tested if atorvastatin’s anticancer effects depended on when the statin treatment commenced. We found that TGF-β-treated epithelial cancer cell lines were more sensitive to atorvastatin. This sensitivity was more pronounced when statin addition preceded TGF-β treatment. We also demonstrated that such sensitivity was even further enhanced by the concomitant downregulation of *HMGCR* expression in cancer cells.

## Results

### TGF-β1 induces markers of EMT initiation in epithelial cancer cell lines

**Figure 1:**
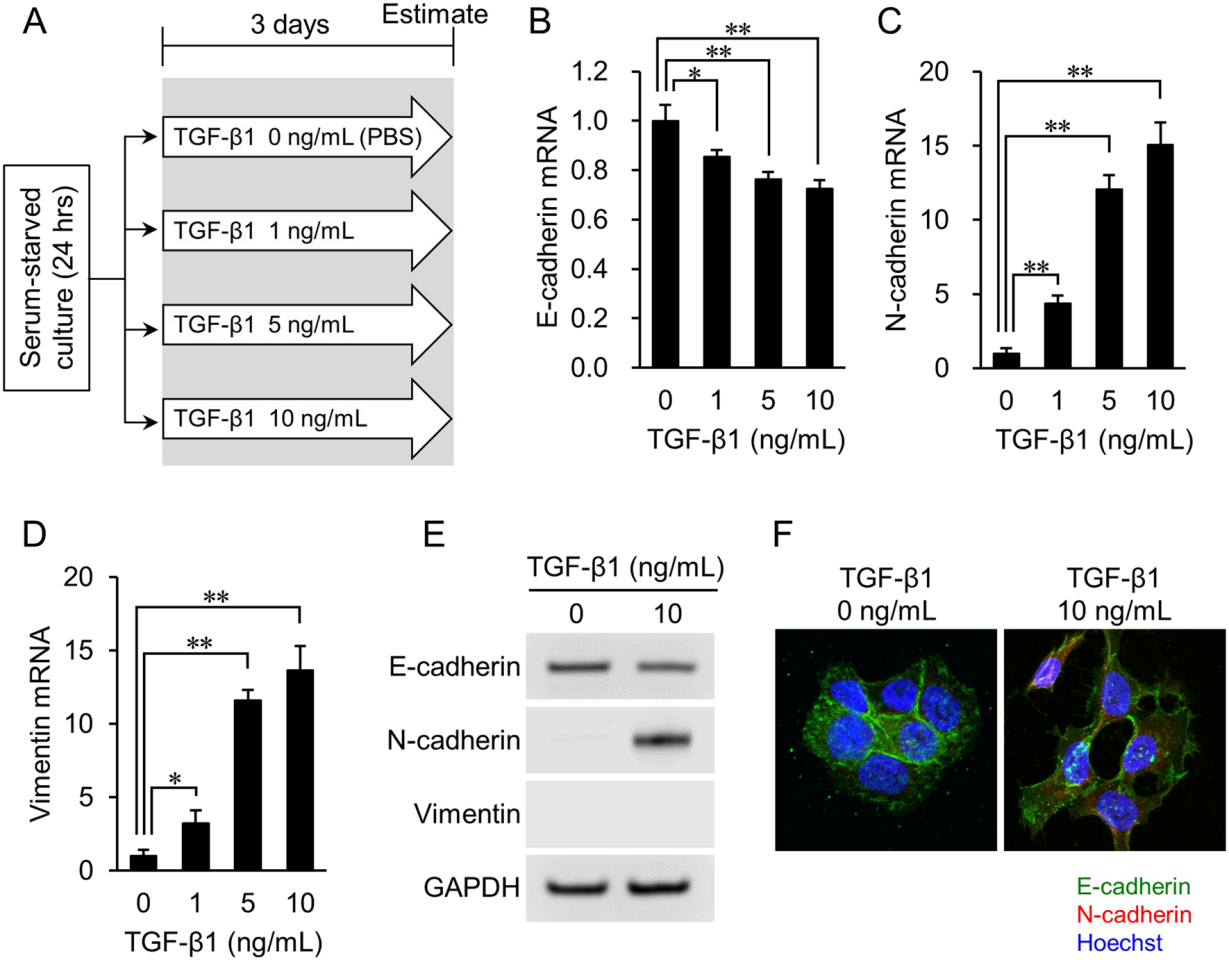
Expression of EMT markers in NCI-H322M cells treated with TGF-β1. (**A**) Overview of experimental procedures. (**B–D**) Quantitative PCR analysis of E-cadherin (**B**), N-cadherin (**C**), and vimentin (**D**) mRNA expression in cells treated with TGF-β1. Data are normalized to 18S rRNA level in each sample and are expressed as values relative to that of the internal control. RNA isolated from cells cultured without TGF-β1 is used as control. The measurement values for each group are compared using the Bonferroni-Dunn *post-hoc* test. Mean ± SD, n = 3, *p < 0.05, **p < 0.01; Comparison with TGF-β1 (−) control (0 ng/mL). (**E**) Protein levels of E-cadherin, N-cadherin, and vimentin are determined by western blot analyses. GAPDH expression is used as a loading control. Representative images from three independent experiments are shown. (**F**) Immunofluorescence staining images of EMT markers. E-cadherin (green), N-cadherin (red), and cell nuclei (blue, Hoechst staining) are shown. N-cadherin expression is only observed in TGF-β1 induced cells.

To examine whether inducing a mesenchymal cell phenotype in epithelial cancer cell lines increased their susceptibility to atorvastatin’s growth-inhibitory effect, we first tested their response to TGF-β1. The experimental design is shown in Fig. 1A. Briefly, lung cancer-derived NCI-H322M cells were serum-starved for 24 h, then incubated with 1, 5, or 10 ng/mL of TGF-β1 for 72 h, as described previously^20–22^.

Real-time PCR analysis showed that the gene expression level of E-cadherin, an epithelial cell marker, significantly decreased in inverse proportion to the concentration of TGF-β1 (*p* < 0.01) (Fig. 1B). In contrast, the gene expression levels of mesenchymal markers N-cadherin (Fig. 1C) and vimentin (Fig. 1D) increased significantly, and proportionally, to the concentration of TGF-β1 (*p* < 0.01). Changes in the protein expression of E-cadherin and N-cadherin were consistent with changes in gene expression. However, vimentin protein levels were not detected (Fig. 1E and Supplementary Fig. S1). Finally, fluorescent immunostaining confirmed that E-cadherin expression had slightly decreased, while N-cadherin was expressed in cells after TGF-β1 treatment (Fig. 1F). Thus, TGF-β1 treatment induced at least some markers of (partial) EMT in NCI-H322M cells.

### TGF-β1 treatment promotes atorvastatin sensitivity in epithelial cancer cell lines

**Figure 2:**
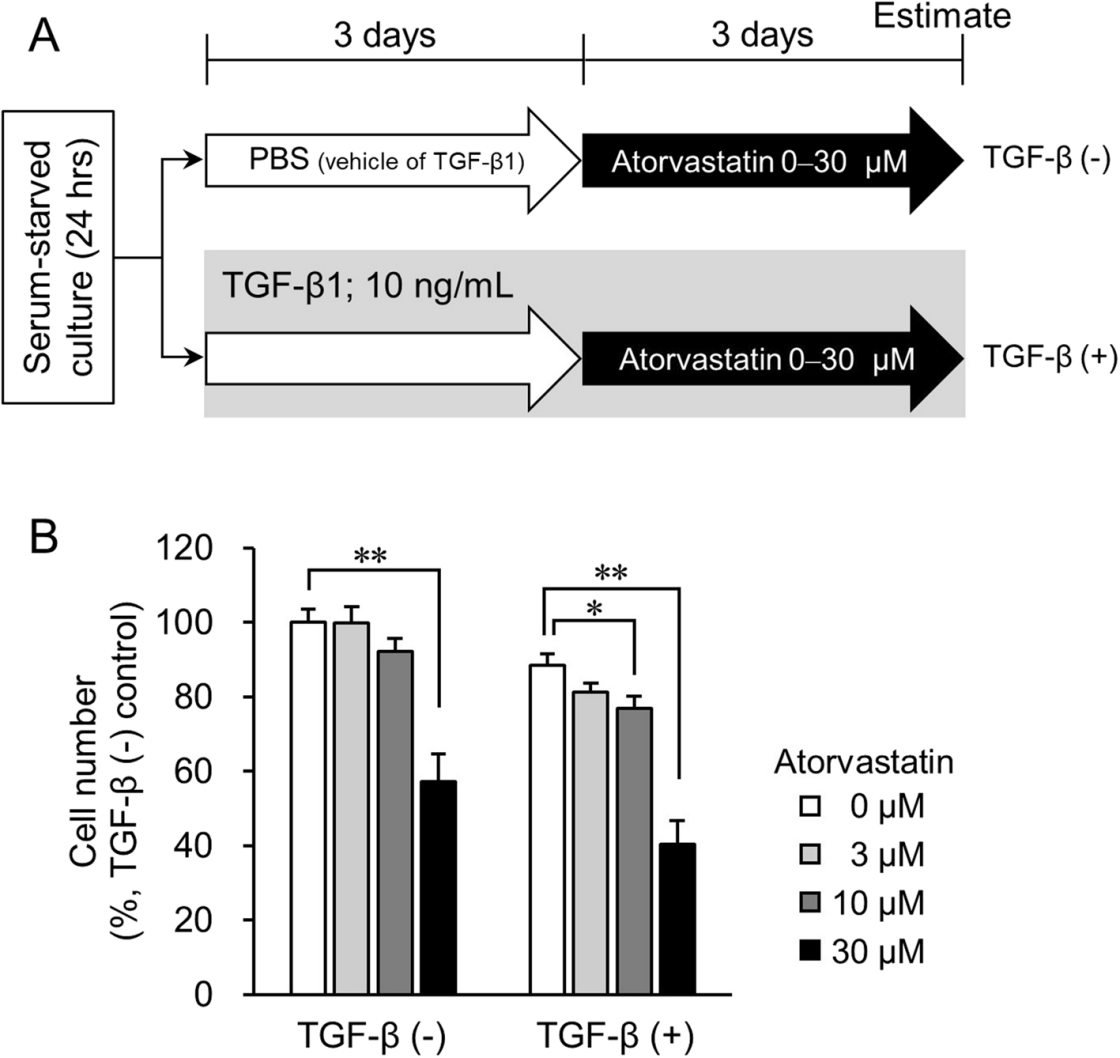
Effect of TGF-β on growth inhibition induced by atorvastatin in NCI-H322M cells. (**A**) Overview of experimental procedures. The group treated with atorvastatin post TGF-β1 induction is designated as TGF-β (+). PBS-treated control is designated as TGF-β (−). (**B**) Cell number in TGF-β (+) group with respect to cell number in TGF-β (−) control cells that was set to 100%. Each value represents mean ± SD (n = 3). Measurement values for each group are compared using the Bonferroni-Dunn *post-hoc* tests. *p < 0.05, **p < 0.01, with respect to each control group.

To further test the sensitivity of NCI-H322M cells to statin treatment we first treated cells with 10 ng/mL TGF-β1. Next, different atorvastatin concentrations were added to the cell culture medium (Fig. 2A, TGF-β (+) group). The proliferation of cells untreated with TGF-β1 (TGF-β (−) group) was only slightly attenuated by 10 µM atorvastatin, but was significantly attenuated at a concentration of 30 µM (*p* < 0.01) (Fig. 2B). In contrast, in the TGF-β (+) cells, the growth of cells significantly decreased at both 10 µM and 30 µM atorvastatin concentrations (*p* < 0.05, and *p* < 0.01, respectively) (Fig. 2B, right). Similar effects were observed in TGF-β1-treated ovarian cancer-derived OVCAR3 cells (Supplementary Fig. S2). In both cell lines, induction with TGF-β1 alone led to a small decline in cell numbers (Fig. 2B and Supplementary Fig. S2B). Thus, TGF-β1 treatment improved the growth inhibitory effects of atorvastatin, to some degree, in epithelial-like cancer cells.

### Atorvastatin pretreatment of epithelial cancer cells increases their sensitivity to statin post TGF-β treatment

**Figure 3:**
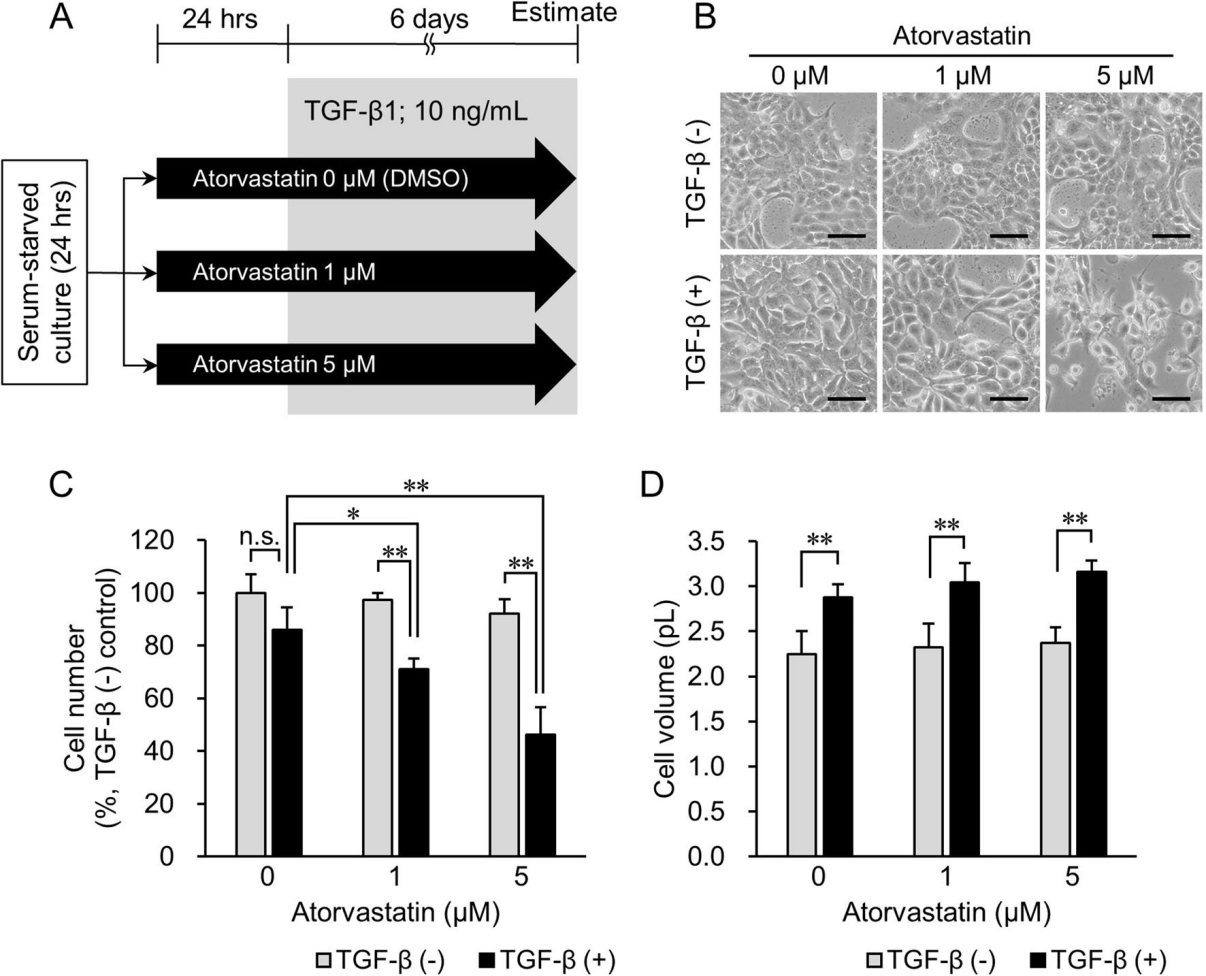
Effect of pretreatment with atorvastatin on cell number and cell volume in the TGF-β1 (−) and TGF-β1 (+) groups. (**A**) Overview of experimental procedures. The group treated with TGF-β1 in the presence of atorvastatin is designated as TGF-β (+) group. Cells treated with PBS are designated as TGF-β (−) group. DMSO-treated cells are used as no-drug treatment controls. (**B**) Phase-contrast imaging of cells treated with 0–5 μM atorvastatin after inducing TGF-β1 signaling. In the TGF-β (−) group (top panel), no significant difference in cell morphology is observed regardless of treatment with atorvastatin. In the TGF-β (+) group (bottom panel), cell number decreased in an atorvastatin dose-dependent manner. Additionally, the cytoplasmic volume of TGF-β (+) cells increased more than that in TGF-β (−) cells. Scale bar = 100 μm. (**C**) Cell number in the TGF-β (−) group and TGF-β (+) group treated with 0–5 μM atorvastatin. Value in TGF-β (−) control cells (atorvastatin 0 μM) is set as 100%. (**D**) Cell volume in the TGF-β (−) group and TGF-β (+) group treated with 0–5 μM atorvastatin. Each value represents mean ± SD (n = 3). Measurement values for each group are compared using the Bonferroni-Dunn *post-hoc* test. *p < 0.05, **p < 0.01, n.s. not significant.

Next, we tested whether atorvastatin pretreatment improved the growth-inhibitory effect on TGF-β1-treated epithelial cancer cells. After serum starvation, NCI-H322M (Fig. 3A) and OVCAR3 (Supplementary Fig. S3A) cells were incubated with and without 1 µM or 5 µM atorvastatin in serum-starved medium for 24 h prior to TGF-β1 treatment for 6 days (TGF-β (+) group). TGF-β1-untreated and atorvastatin-treated cells served as the control (TGF-β (−)) group. The difference in cell number between the TGF-β (+) and TGF-β (−) groups at 0 µM atorvastatin concentration (Fig. 3C) was statistically not significant at the end of the experiment. However, at an increased concentration of atorvastatin, the cell numbers more significantly decreased in the TGF-β (+) group than those of the TGF-β (−) group (*p* < 0.01) receiving the same atorvastatin dose (Fig. 3C). Similar effects were observed in TGF-β1-treated OVCAR3 cells (Supplementary Fig. S3B).

In previous studies, TGF-β1 treatment was shown to increase cell volume by activating the mammalian target of rapamycin (mTOR) pathway, which in turn was inhibited by rapamycin treatment^23^. Therefore, we also tested cell volume changes. The cell volume of NCI-H322M cells in the TGF-β (+) group grew to approximately 1.3 × that of cells in the TGF-β (−) group, regardless of atorvastatin treatment (*p* < 0.01) (Fig. 3B,D). This suggests that atorvastatin pretreatment did not affect cell growth via mTOR signaling.

**Figure 4:**
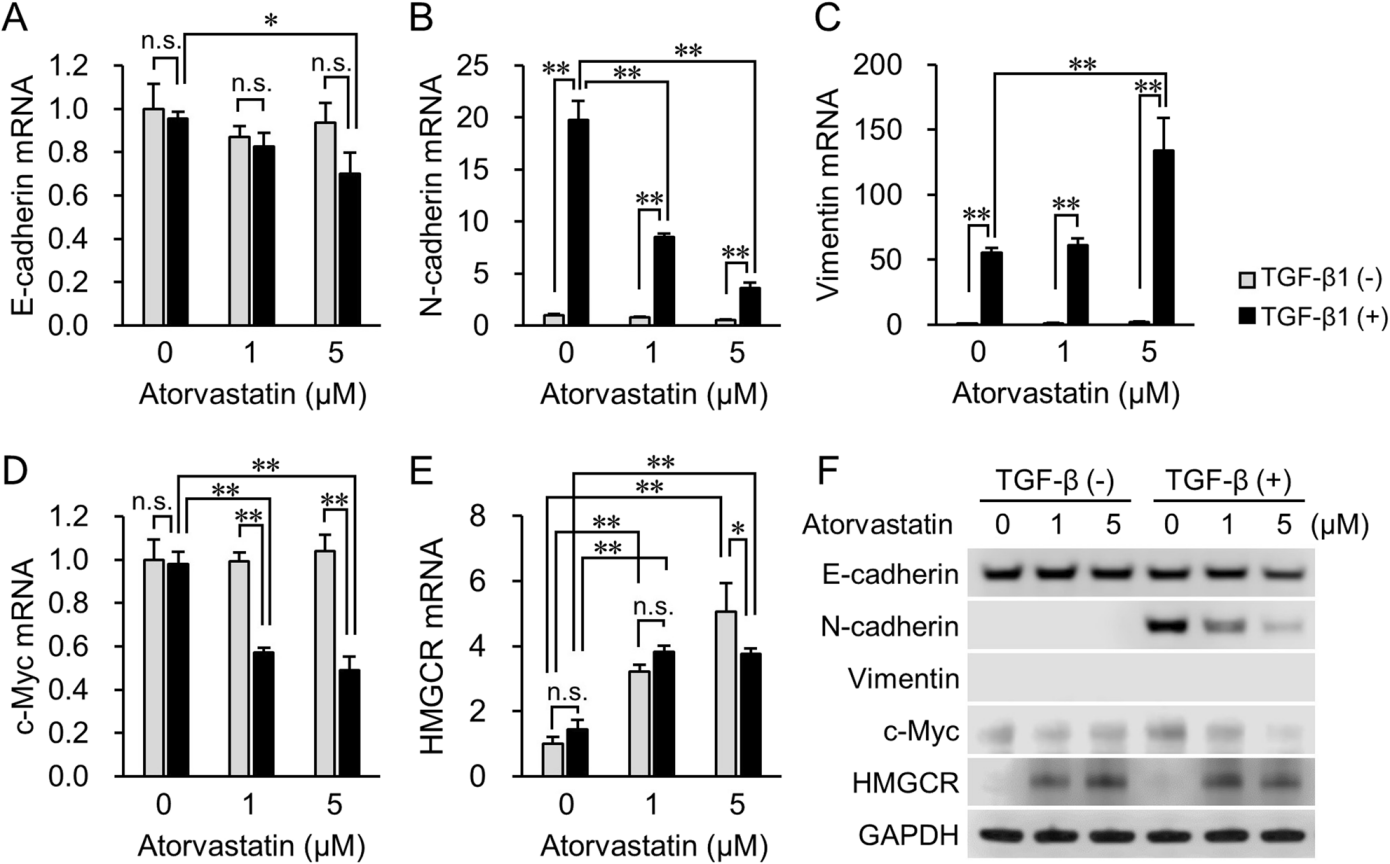
mRNA and protein expression levels of EMT-related molecules, regulator of cell proliferation, and target of statins in TGF-β (−) and TGF-β (+) groups. Real-time PCR analyses of expression of (**A**) E-cadherin, (**B**) N-cadherin, (**C**) vimentin, (**D**) c-Myc, and (**E**) HMGCR genes after the start of TGF-β1 incubation. Cells incubated with PBS are designated as TGF-β1 (−). Data are normalized to 18S rRNA levels in each sample and expressed as values relative to those of the internal control. The measurement values for each group are compared using the Bonferroni-Dunn *post-hoc* test. Mean ± SD, n = 3, *p < 0.05, **p < 0.01, n.s. not significant. (**F**) Western blot analysis of the expression of E-cadherin, N-cadherin, vimentin, c-Myc, and HMGCR after induction with TGF-β1. GAPDH expression is used as the loading control. Representative images from three independent experiments are shown.

At the end of the experiment (Fig. 3A), we also tested how EMT markers’ expression changed in atorvastatin’s presence, both before and after TGF-β1 treatment (Fig. 4 and Supplementary Fig. S4, S5). E-cadherin expression remained essentially unchanged at both the mRNA (Fig. 4A) and protein levels (Fig. 4F and Supplementary Fig. S4A) in both the presence and absence of TGF-β1 at a low (1 µM) atorvastatin dose. However, treatment with 5 μM atorvastatin slightly increased TGF-β1-induced downregulation of E-cadherin expression (Fig. 4A,F). Moreover, an increased atorvastatin concentration strongly inhibited TGF-β1-induced upregulation of N-cadherin expression, both at the mRNA and protein levels (Fig. 4B,F and Supplementary Fig. S4B). Treatment with 5 μM atorvastatin significantly accelerated TGF-β1-induced vimentin mRNA expression (Fig. 4C) but no vimentin protein expression was detected (Fig. 4F and Supplementary Fig. S4C). In the TGF-β (−) group, there was no significant difference in the expression of the cell cycle regulator, c-Myc, at both the mRNA and protein levels, whereas atorvastatin attenuated c-Myc expression in the TGF-β (+) group at all tested drug concentrations (Fig. 4D,F and Supplementary Fig. S4D). Furthermore, atorvastatin treatment increased the mRNA expression of the statin target enzyme, *HMGCR*, in a dose-dependent manner in both the TGF-β (−) and TGF-β (+) groups (Fig. 4E). In contrast, after the addition of 5 μM atorvastatin, there was a decline in the induction of *HMGCR* gene expression in the TGF-β1-treated cells (Fig. 4E). These findings paralleled protein level changes; atorvastatin treatment strongly induced HMGCR protein expression (Fig. 4F), yet in TGF-β1-induced cells exposed to 5 μM atorvastatin this effect was weaker (Fig. 4F, Supplementary Fig. S4E).

### Attenuated HMGCR expression promotes sensitivity to atorvastatin in TGF-β-treated epithelial cancer cells

**Figure 5:**
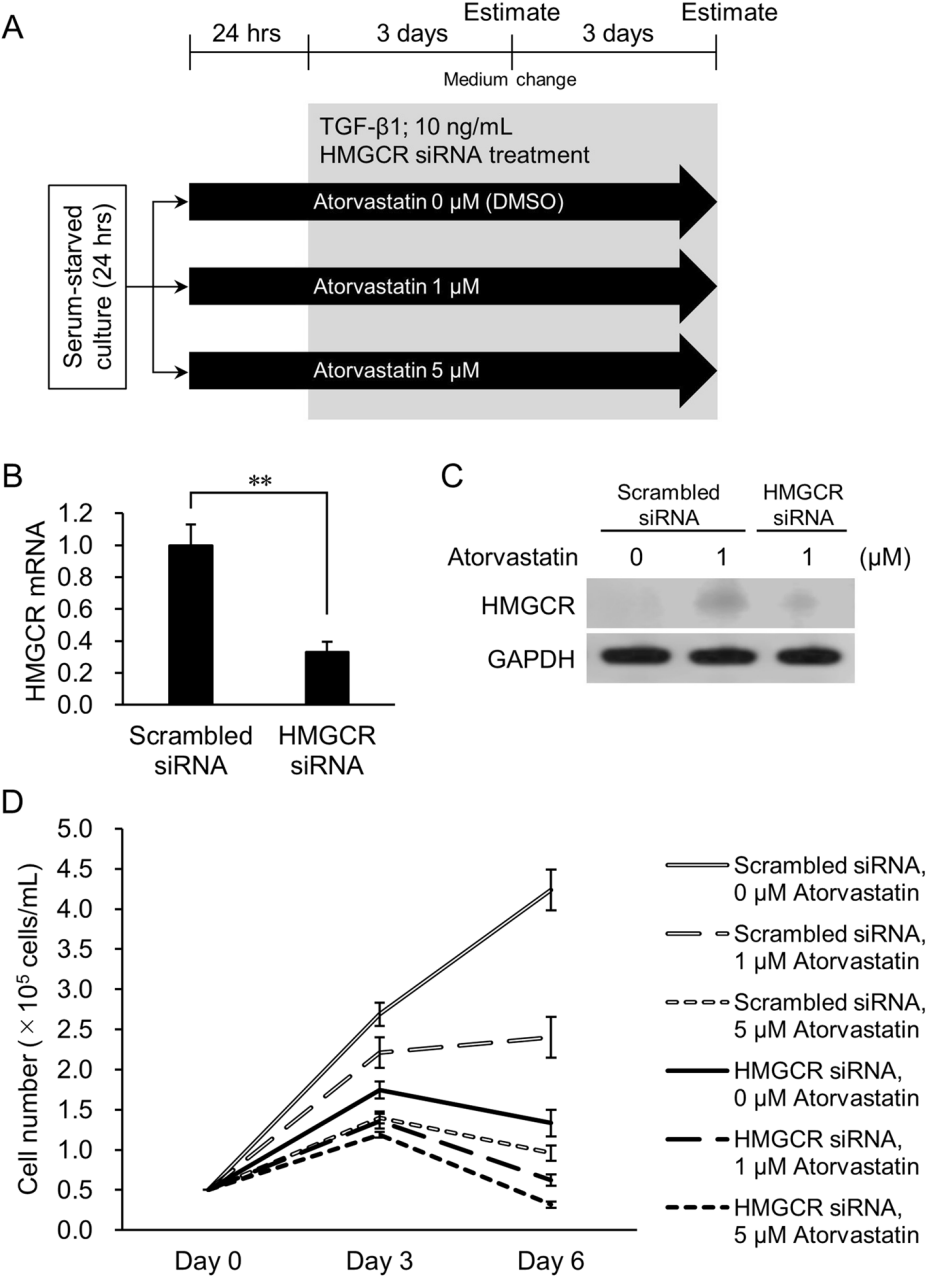
Effect of *HMGCR* knockdown on atorvastatin-pretreated and TGF-β-induced NCI-H322M cells. (**A**) Overview of experimental procedures. Cells were treated with TGF-β1 in the presence of 1 μM or 5 μM atorvastatin and *HMGCR* siRNA. Scrambled siRNA was used as negative control for RNAi experiment. Cell numbers were counted at days 3 and 6 after TGF-β1 induction. (**B**) Expression of *HMGCR* mRNA in cells 3 days after transfection of *HMGCR* siRNA. Data are normalized to 18S rRNA level in each sample and are expressed as values relative to that of the internal control. The measurement values for each group are compared using student’s two-tailed *t*-test. Mean ± SD, n = 3, **p < 0.01. (**C**) HMGCR protein levels in cells 3 days after transfection of *HMGCR* siRNA. Panel indicates western blot analysis of HMGCR after siRNA transfection in cells. (**D**) Cell number of the TGF-β1 (+) group treated with 0–5 μM atorvastatin and *HMGCR* siRNA at days 3 and 6. Each value represents mean ± SD (n = 3). Statistical analysis is shown in Supplementary Table S1.

Downregulation of HMGCR activity contributes to statins’ anticancer effect by inhibiting post-translational modification (prenylation) of small G-proteins (e.g., Ras, Rho, Rac, and Cdc42)^3^. We previously showed that siRNA-mediated attenuation of HMGCR expression enhanced the growth and proliferation-inhibitory effect of atorvastatin on relatively statin-resistant NCI-H322M epithelial lung cancer cells^24^. Therefore, we next tested whether downregulating the expression of HMGCR further enhanced statin’s inhibitory effect in atorvastatin-pretreated, TGF-β1-treated NCI-H322M cells. The experimental design is shown in Fig. 5A. Briefly, NCI-H322M cells were treated with 1 μM or 5 μM atorvastatin for 24 h. EMT was then induced with 10 ng/mL TGF-β1 for 6 days, alongside *HMGCR* siRNA treatment. Downregulation of HMGCR expression, observed at both the mRNA (Fig. 5B) and protein levels (Fig. 5C and Supplementary Fig. S6), enhanced atorvastatin’s inhibitory effects at both the 1 and 5 μM concentrations (Fig. 5D; Supplementary Fig. S7; Supplementary Table S1). Thus, TGF-β1-treated NCI-H322M cells with reduced HMGCR expression were more sensitive to atorvastatin’s inhibitory effects than those with uninhibited HMGCR expression.

## Discussion

EMT is a stepwise cellular transdifferentiation process from epithelial to mesenchymal state^25^. EMT plays an important role in several physiological and pathological processes such as wound healing and metastasis formation^12,13,26^. EMT-induced initiation of the metastatic cascade is intimately related to the tumor microenvironment’s state^26^. For example, senescent fibroblasts often acquire a proinflammatory state characterized by their secretion of extracellular proteases, growth factors, and cytokines^27^. Some of these factors, such as brain-derived neurotrophic factor (BDNF) and TGF-β1, can promote EMT-like phenotypes, in both normal cells^28–30^ and in tumor cells bearing mutations in oncogenes and tumor suppressor genes^14,31^.

TGF-β1 initiates EMT by activating Smad2/3. This is followed by increased expression of transcription factors such as ZEB1, which ordinarily suppresses the expression of E-cadherin^12,13,32^. Intracellular signals involved in the expression of E-cadherin are thereby altered, leading to the expression of mesenchymal markers like N-cadherin and vimentin^33^.

We previously reported that the epithelial lung cancer cell line NCI-H322M had high E-cadherin expression and very low or no vimentin expression^4^. Because statins exert anticancer effects most potently on mesenchymal-like cancer cells^4^, we hypothesized that cancer cells that acquire at least some mesenchymal properties become more sensitive to statins. However, atorvastatin treatment after TGF-β1 treatment only slightly affected the proliferation of NCI-H322M and OVCAR3 cells. In contrast, atorvastatin pretreatment significantly attenuated cell proliferation in the TGF-β1-treated group in a dose-dependent manner. Expression levels of c-Myc are minimal in quiescent cells in vitro; however, once the cells are exposed to mitogenic stimuli, c-Myc mRNA and protein levels rapidly increase, and the cells enter the G1 phase of the cell cycle^34,35^. A previous study has reported that statins upregulate miR-33b expression and adversely affect c-Myc expression and function in cancer cells^36^. It is possible that the effect of atorvastatin on c-Myc expression differs between TGF-β-treated and untreated cancer cells.

Several studies have reported that statins suppress EMT. Nishikawa *et al.* (2019)^37^ demonstrated that NCI-H1975 lung cancer cells with the EMT-inducible mutant *TP53* (R273H) restored E-cadherin expression and decreased vimentin expression in a simvastatin dose-dependent manner. Fan *et al.* (2016)^38^ similarly reported that atorvastatin treatment effectively negated the TGF-β1-stimulated (1 ng/mL) downregulation of E-cadherin and upregulation of vimentin in A549 lung cancer cells. In our study, atorvastatin pretreatment significantly suppressed the TGF-β1 treatment-induced increase in N-cadherin expression at both the mRNA and protein levels. This effect was inversely proportional to atorvastatin concentration. Loss or reduction in the expression of E-cadherin and upregulation of N-cadherin (the so-called “cadherin switch”) are common features of full- or partial EMT. Functional E-cadherin expression inhibits cell migration, whereas aberrant N-cadherin expression enhances cell migration and invasion in cancer cells regardless of E-cadherin expression^39^. Furthermore, activated TGF-β signaling pathway can induce the expression of cell-surface glycoprotein CD146, a member of the immunoglobulin superfamily. A CD146/ERK cascade can enhance N-cadherin expression during TGF-β-induced EMT^40^. However, statins and farnesyl transferase inhibitors are known to significantly reduce ERK phosphorylation in non-small cell lung cancer cells^41^. It is thus likely that the reduction in N-cadherin expression this study observed occurred due to the inhibition of the CD146/ERK cascade, although the detailed mechanism remains unclear.

Jiang *et al.* (2018)^42^ demonstrated that statins both slightly upregulated *HMGCR* mRNA levels and increased HMGCR protein levels more than tenfold, mainly by preventing ubiquitination and degradation of HMGCR. Indeed, statin-induced upregulation of HMGCR expression appeared to be a common homeostatic reaction in cells^43^. In this study, we found that inducing a partial mesenchymal state did not affect *HMGCR* (mRNA) expression. However, HMGCR protein levels strongly increased in response to statin treatment, although they were slightly lower in cells after TGF-β1 treatment than without it (Fig. 4F, Supplementary Fig. S4E). This effect was countered by siRNA knockdown of the *HMGCR* gene and protein expression (Fig. 5B,C). In turn, this led to a much stronger attenuation of cell growth and proliferation than atorvastatin treatment alone (Fig. 5D).

**Figure 6:**
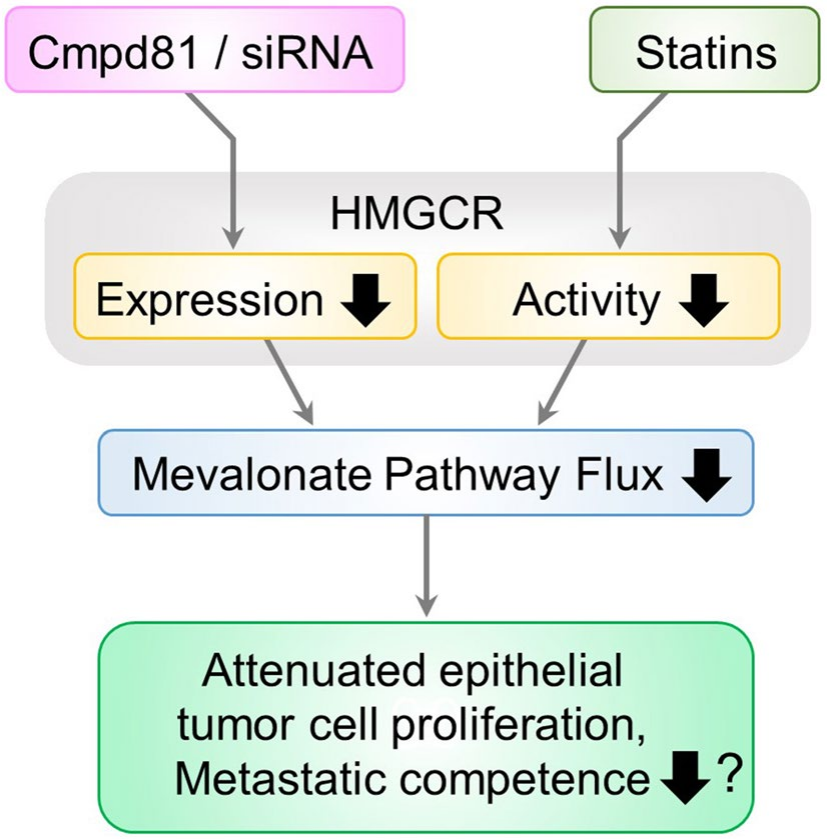
Dual inhibition of HMGCR in TGF-β-induced epithelial cancer cells. Following microenvironment-induced phenotypic state switch in epithelial cancer cells the dual inhibition of HMGCR, by downregulation of its expression (by siRNA or Cmpd81) and its enzymatic activity (by statins) leads to reduced cholesterol levels and reduced synthesis of the mevalonate pathway’s intermediate metabolites that are required for prenylation of signaling proteins. This dual treatment regimen may also reduce the metastatic competence of emerging (micro)tumors to a greater extent than atorvastatin treatment alone^19^.

Altogether, our study demonstrates that inducing phenotypic state switch in epithelial cancer cells and simultaneously downregulating HMGCR expression improves atorvastatin-induced attenuation of cell proliferation. We also found a consistent correlation between atorvastatin treatment-mediated attenuation of TGF-β1-induced- and mesenchymal-like cell proliferation in vitro^4,18^ and metastasis formation *in vivo*^19^. The data of Ishikawa *et al.* (2018)^24^ and Göbel *et al.* (2019)^44^, combined with our current results, moreover demonstrates that attenuating *HMGCR* expression further enhances atorvastatin’s effect in vitro. Indeed, dual treatment with statin and the HMGCR degrader Cmpd81 synergistically decreased LDL-cholesterol levels (and reduced atherosclerotic plaque formation) in a murine model^42^. Therefore, we can hypothesize that the dual inhibition of HMGCR by, on the one hand inhibiting HMGCR activity by statins and, on the other, attenuating *HMGCR* expression by siRNA or Cmpd81 would also delay metastasis formation to a greater extent than atorvastatin treatment alone^19^ (Fig. 6).

## Methods

### Cell culture

We selected two cell lines from the NCI-60 cancer cell panel obtained from the DCTD Tumor Repository (National Cancer Institute, Frederick, MD, USA): bronchoalveolar adenocarcinoma-derived NCI-H322M cells, and ovarian adenocarcinoma-derived OVCAR3 cells. Cells were cultured in RPMI 1640 medium (Thermo Fisher Scientific, Waltham, MA, USA) supplemented with 10% fetal bovine serum (FBS; Biosera, Boussens, France) and penicillin–streptomycin solution (Fujifilm Wako Pure Chemical, Osaka, Japan; final concentration: 100 units/mL penicillin G and 100 μg/mL streptomycin) in a humidified incubator at 37 °C under 5% CO_2_.

For TGF-β induction, 5 × 10^4^ cells/mL were seeded in 12-well plates. After attachment, the cells were incubated with serum-starved medium (0.5% FBS) for 24 h to remove residual TGF-β1 from the media. Cells were then incubated with 1, 5, or 10 ng/mL of recombinant human TGF-β1 in serum-starved medium (Peprotech, Rocky Hill, NJ, USA) for 72 h, as described previously^20–22^. Changes in gene and protein expression levels of epithelial (E-cadherin) and mesenchymal (N-cadherin and vimentin) cell markers were analyzed.

For atorvastatin treatment, cells were treated with 0–30 µM atorvastatin (Sigma-Aldrich, St. Louis, MO, USA) diluted in DMSO (Fujifilm Wako Pure Chemical) under various experimental conditions (Figs. 2A, 3A, and 5A). Equal volumes of DMSO (< 0.1%) served as negative controls.

For some experiments (Fig. 3A), the culture medium was changed to fresh serum-starved medium supplemented with 10 ng/mL TGF-β1 and 0–5 µM atorvastatin. To maintain optimal culture conditions, serum-starved medium containing TGF-β1 and atorvastatin was replaced every 3 days. The cell number was counted after 6 days of incubation. Total RNA and protein were extracted from triplicate samples accordingly.

In siRNA experiments (see below), after 24 h of serum starvation, NCI-H322M cells were treated with atorvastatin at concentrations of 1 μM or 5 μM for 24 h. Cells were then treated with 10 ng/mL TGF-β1 for 6 days in the presence of 1 μM or 5 μM atorvastatin and *HMGCR* siRNA (Fig. 5A). Scrambled siRNA was used as a negative control for RNAi experiments. Cell numbers were counted on days 3 and 6 after TGF-β1 addition.

### Downregulation of HMGCR expression by siRNA

Predesigned siRNA oligonucleotides specific to *HMGCR* (NM_000859, siRNA ID#s142, targeted exon 12, siRNA location: 1698) were obtained from Thermo Fisher Scientific. Silencer negative control siRNA (#4390843, Thermo Fisher Scientific) was used as a scrambled siRNA. This sequence, provided by the manufacturer, showed no significant homology to any gene. Reverse transfections were performed in 12-well dishes (5 × 10^4^ cells/mL) according to the manufacturer's instructions using Lipofectamine RNAiMax (Thermo Fisher Scientific), Opti-MEM (Thermo Fisher Scientific), and siRNAs (final concentration, 10 nM) for the respective targets. Atorvastatin-treated NCI-H322M cells were harvested on days 3 and 6 after transfection to analyze cell viability. Transfection efficiency was assessed by quantitative reverse transcription polymerase chain reaction (RT-PCR) and western blotting. The PCR primer set, which included siRNA target sites, was as follows: sense primer 5′-CCCAGCCTACAAGTTGGAAA-3′ and anti-sense primer 5′-AACAAGCTCCCATCACCAAG-3′ (PCR product: 152 bp).

### Real-time PCR

An RNeasy mini kit (Qiagen, Hilden, Germany) was used to extract total RNA from the cells. cDNA was synthesized from 1 μg of total RNA using ReverTra Ace qPCR RT with gDNA Remover kit (Toyobo, Osaka, Japan). The primer sets used for PCR are shown in Supplementary Table S2. PCR was performed using LightCycler FastStart DNA MasterPLUS SYBR Green I mix and a LightCycler rapid thermal cycler system (Roche Diagnostics, Lewes, UK).

### Western blotting

Cells in a petri dish were washed twice with cold PBS, followed by incubation with CelLytic M solution (Sigma-Aldrich) on ice for 5 min. Subsequently, the cells were scraped and collected into 1.5-mL microfuge tubes and homogenized by passing through a 27-gauge syringe needle. The cell lysates were centrifuged at 16,000 × g at 4 °C for 15 min, and the supernatants were transferred to new tubes. Protein concentrations were measured by the bicinchoninic acid (BCA) method using the BCA Protein Assay Kit (Thermo Fisher Scientific). Proteins were incubated at 90 °C for 3 min with Laemmli sample buffer (Bio-Rad, Hercules, CA, USA) prior to electrophoresis. NuPAGE® 4%–12% Bis-Tris gel (Thermo Fisher Scientific) was used for electrophoresis, and 10 μg of protein lysates were loaded per lane. After electrophoresis, the proteins were transferred onto a nitrocellulose membrane using iBlot® Gel Transfer Stacks Nitrocellulose (Thermo Fisher Scientific) and an iBlot® Gel Transfer Device (Thermo Fisher Scientific). After blocking the nitrocellulose membrane with 5% (w/v) skim milk (Morinaga Milk Industry, Tokyo, Japan), the membranes were incubated with primary antibodies according to the manufacturer's instructions. Anti-E-cadherin rabbit monoclonal antibody (1:1000 dilution, 24E10; Cell Signaling Technology, Beverly, MA, USA), anti-N-cadherin mouse monoclonal antibody (1:1000 dilution, 610920; BD Biosciences, San Diego, CA, USA), and anti-vimentin mouse monoclonal antibody (1:1000 dilution, 5G3F10; Cell Signaling Technology) were used as primary antibodies to detect EMT markers. Protein levels of c-Myc and HMGCR were determined using anti-c-Myc rabbit monoclonal antibody (1:1000 dilution, E5Q6W; Cell Signaling Technology) and anti-HMGCR rabbit monoclonal antibody (1:1000 dilution, AMab90618; Atlas, Cambridge, UK), respectively. Anti-glyceraldehyde-3-phosphate dehydrogenase (GAPDH) rabbit monoclonal antibody (1:1000 dilution, 14C10; Cell Signaling Technology) was used to detect GAPDH as an internal standard. After washing the nitrocellulose membranes with Tris buffer, they were incubated with horseradish peroxidase (HRP)-labeled secondary antibodies (anti-mouse IgG goat antibody; R&D Systems, Minneapolis, MN, USA) or anti-rabbit IgG goat antibody (SeraCare, Milford, MA, USA) for 1 h. The nitrocellulose membrane was washed again and incubated with Clarity Western ECL substrate chemiluminescent detection reagent (Bio-Rad) for 5 min. Protein signals were detected using a C-DiGit Blot Scanner (Li-Cor Biosciences, Lincoln, NE, USA).

### Immunofluorescence microscopy

Cultured NCI-H322M cells grown on coverslips in a 24-well plate were fixed with 2% paraformaldehyde (Nacalai Tesque, Kyoto, Japan) for 30 min, washed with PBS, and permeabilized with 0.1% Triton-X-100 (Thermo Fisher Scientific) in PBS for 15 min. Following a PBS wash, non-specific proteins were blocked with 2% BSA (Fujifilm Wako Pure Chemical) for 15 min. The cells were incubated with a mixture of two primary antibodies: anti-E-cadherin rabbit monoclonal antibody (1:200 dilution, 24E10; Cell Signaling Technology) and anti-N-cadherin mouse monoclonal antibody (1:200 dilution, 610920; BD Biosciences) for 1 h at RT. Coverslips were washed with PBS and incubated with CF®488A goat anti-rabbit IgG (1:200 dilution, Biotium, Hayward, CA, USA) and CF®568 goat anti-mouse IgG (1:200 dilution, Biotium) antibodies in the dark for 15 min. Following a PBS wash, nuclei were stained with Hoechst 33342 (5 μg/mL; Nacalai Tesque) for 15 min. The cells were then washed and mounted in an aqueous-based mounting medium, ClearMount™ (Thermo Fisher Scientific). Images were captured with a 60 × oil objective lens on an Olympus Fluoview FV10i confocal microscope (Olympus, Tokyo, Japan).

### Cell number and cell volume measurement

NCI-H322M and OVCAR3 cells cultured in 12-well plates were washed with PBS and detached with 0.05% trypsin/EDTA (Fujifilm Wako Pure Chemical). Trypsin was inactivated by adding complete medium. The cells were counted using a Scepter handheld automated cell counter (Millipore, Billerica, MA, USA). The ratio of the number of cells in each experimental group to the average number in the control group was considered as the actual cell number and survival rate of the control group was defined as 100%.

### Statistical analyses

Statistical analyses were performed using Excel Statistics 2016 for Windows (version 3.21; SSRI, Tokyo, Japan). The data were compared using Student’ s two-tailed *t*-test and one-way or two-way analysis of variance, followed by Bonferroni-Dunn *post-hoc* tests, with a significance level of *p* < 0.05.

## Supplementary Information

Supplementary Information.
