# Supplementary material for: Concomitant attenuation of HMGCR expression and activity enhances the growth inhibitory effect of atorvastatin on TGF-β-treated epithelial cancer cells
Source: Sci Rep. 2021 Jun 17;11:12763. doi: 10.1038/s41598-021-91928-3 (PMC8211663; doi:10.1038/s41598-021-91928-3)
Supplement: Supplementary file 1 — Supplementary Information. [file 41598_2021_91928_MOESM1_ESM.pdf]

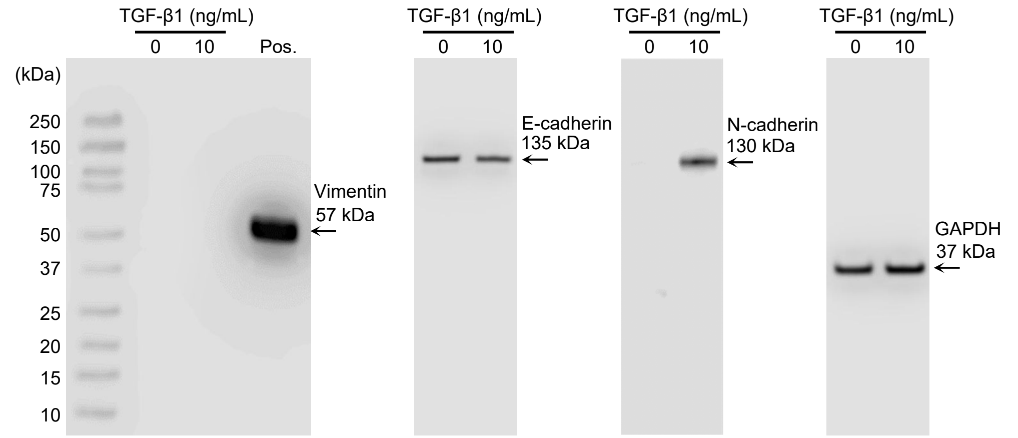

Fig. S1. Western blot analysis of extracts from NCI-H322M cells treated with TGF-β1 for 3 days

All western blot images are full-length blots of vimentin, E-cadherin, N-cadherin, and GAPDH. Mesenchymal-like cancer cell line HOP-92 was used as positive control to detect vimentin protein. The membranes were stripped with Western Blot Stripping Buffer (Takara) for reprobing with other antibodies.

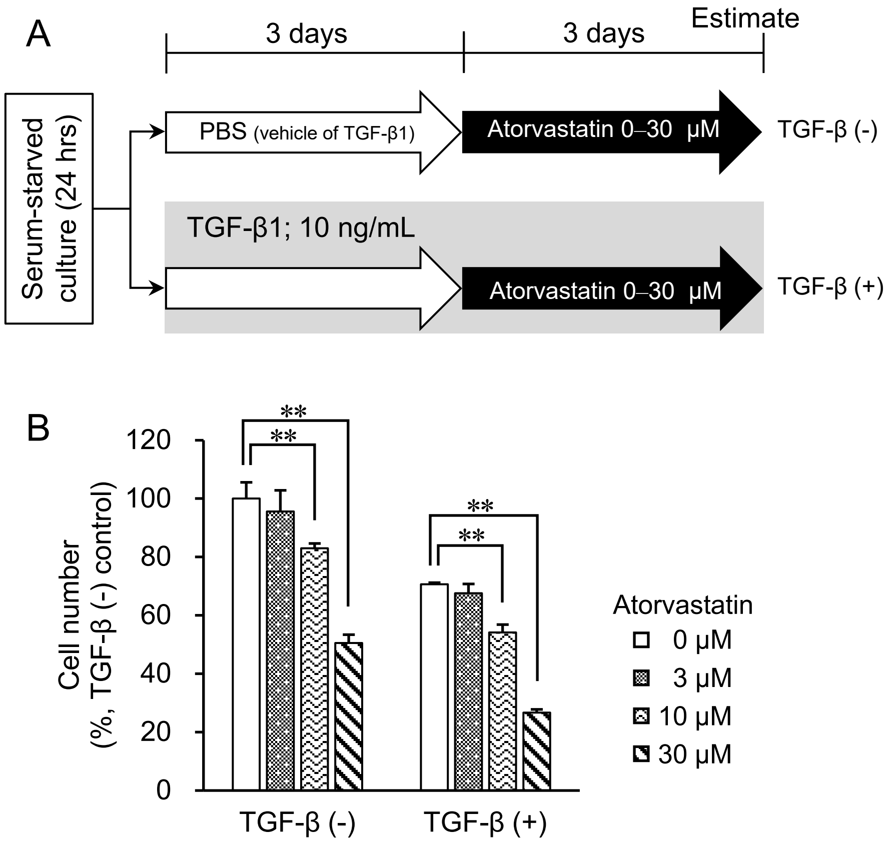

Fig. S2. Effect of TGF-β on growth inhibition induced by atorvastatin in OVCAR3 cells

(A) Overview of experimental procedures. The group treated with atorvastatin post TGF-β1 induction is designated as TGF-β (+). PBS-treated control is designated as TGF-β (-). **(B)** Cell number in TGF-β (+) group with respect to cell number in TGF-β (-) control cells that was set to 100%. Each value represents mean ± SD (n=3). Measurement values for each group are compared using the Bonferroni-Dunn *post-hoc* tests. **p<0.01, with respect to each control group.

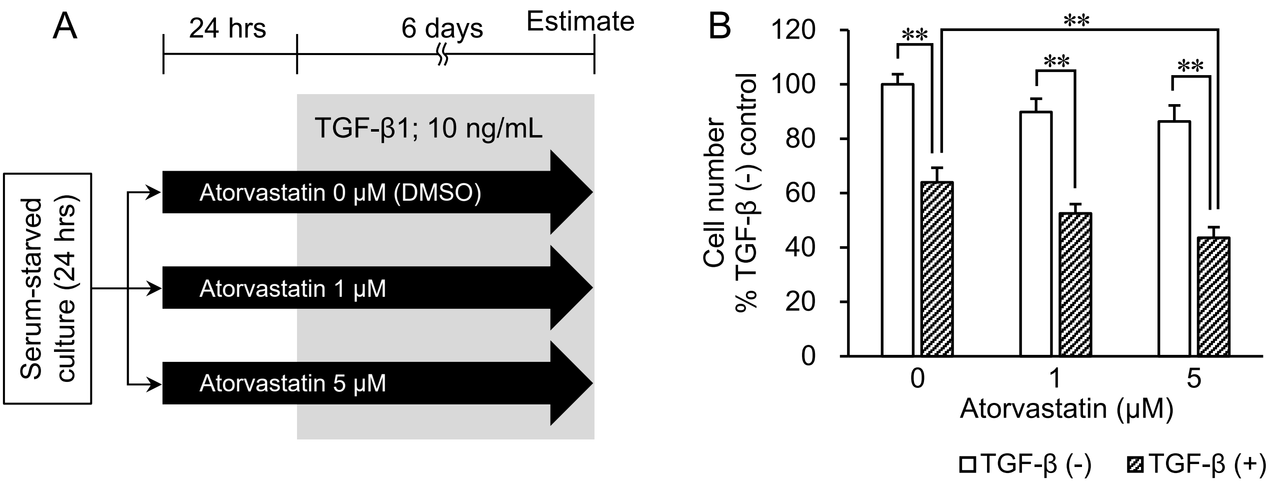

Fig. S3. Effect of pretreatment with atorvastatin on cell number in the TGF-β1 (-) and TGF-β1 (+) groups of OVCAR3 cells

(A) Overview of experimental procedures. The group treated with TGF-β1 in the presence of atorvastatin is designated as TGF-β1 (+) group. Cells treated with PBS are designated as TGF-β1 (-) group. DMSO-treated cells are used as no-drug treatment controls. **(B)** Cell number in the TGF-β1 (-) group and TGF-β1 (+) group treated with 0–5 μM atorvastatin. Value in TGF-β1 (-) control cells (atorvastatin 0 μM) is set as 100%. Each value represents mean ± SD (n=3). Measurement values for each group are compared using the Bonferroni-Dunn *post-hoc* test.**p<0.01

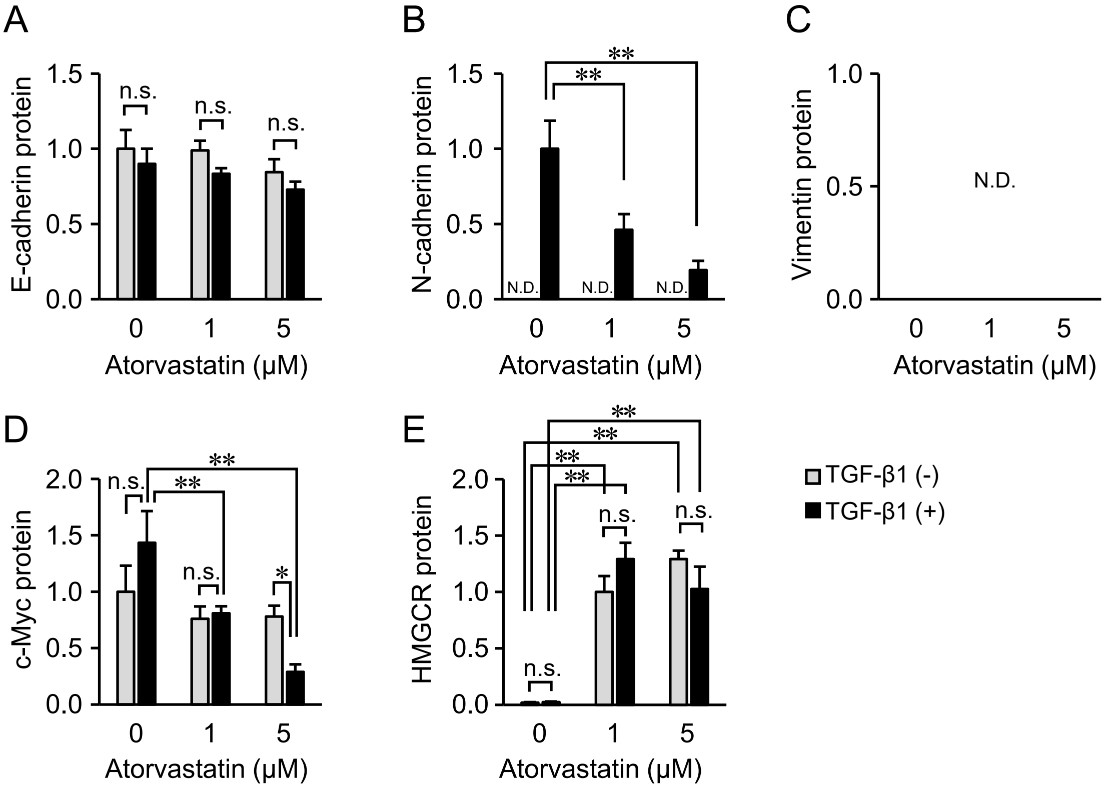

Fig. S4. Quantification of protein expression on EMT-related molecules, regulator of cell proliferation, and target of statins in TGF-β1 (-) and TGF-β1 (+) groups

(A) E-cadherin, **(B)** N-cadherin, **(C)** vimentin, **(D)** c-Myc, and **(E)** HMGCR protein expression are analyzed after 6 days of TGF-β1-incubation. Each band's intensity on the western blot was quantified by ImageJ software (NIH). Data were normalized to GAPDH protein level in each sample and were expressed as values relative to that of internal control. Measurement values for each group are compared using the Bonferroni-Dunn *post-hoc* test. Mean ± SD, n=3, *p<0.05, **p<0.01, n.s. not significant, N.D. not detected.

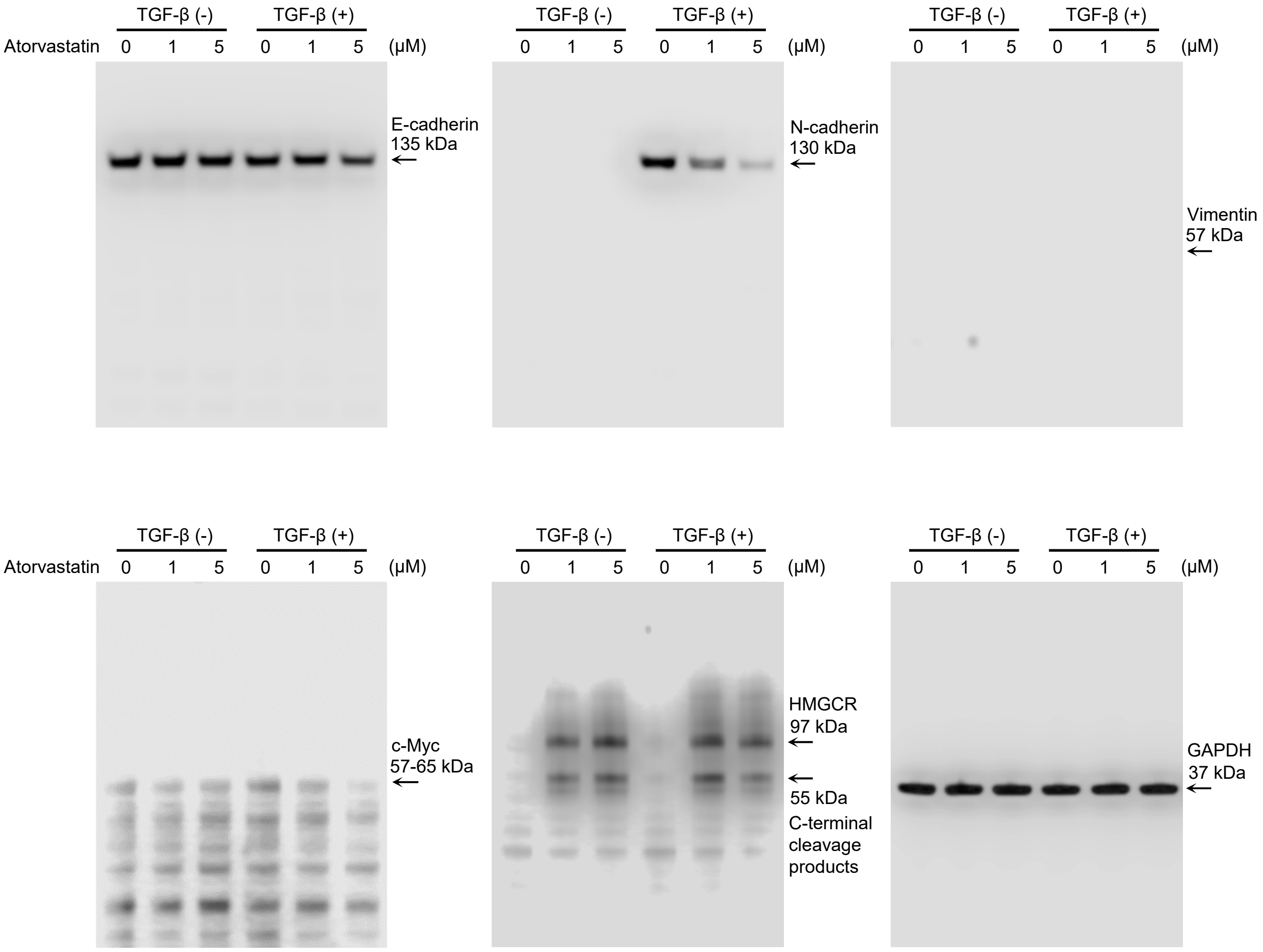

Fig. S5. Western blot analyses in TGF-β1 (-) and TGF-β1 (+) groups

All western blot images are full-length blots of E-cadherin, N-cadherin, vimentin, c-Myc, HMGCR, and GAPDH. The membranes were stripped with Western Blot Stripping Buffer (Takara) for reprobing with other antibodies.

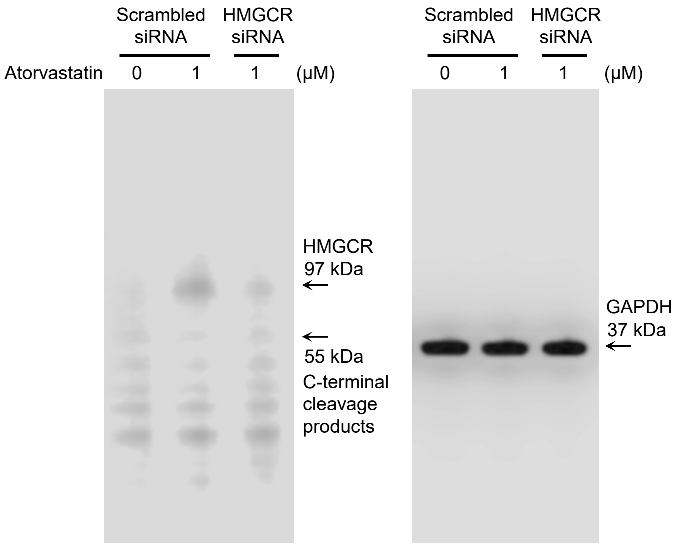

Fig. S6. Western blot analyses of extracts from NCI-H322M cells after transfection of HMGCR siRNA.

All western blot images are full-length blots of HMGCR and GAPDH. Scrambled siRNA is used as negative control for RNAi experiment. The membranes were stripped with Western Blot Stripping Buffer (Takara) for reprobing with other antibodies.

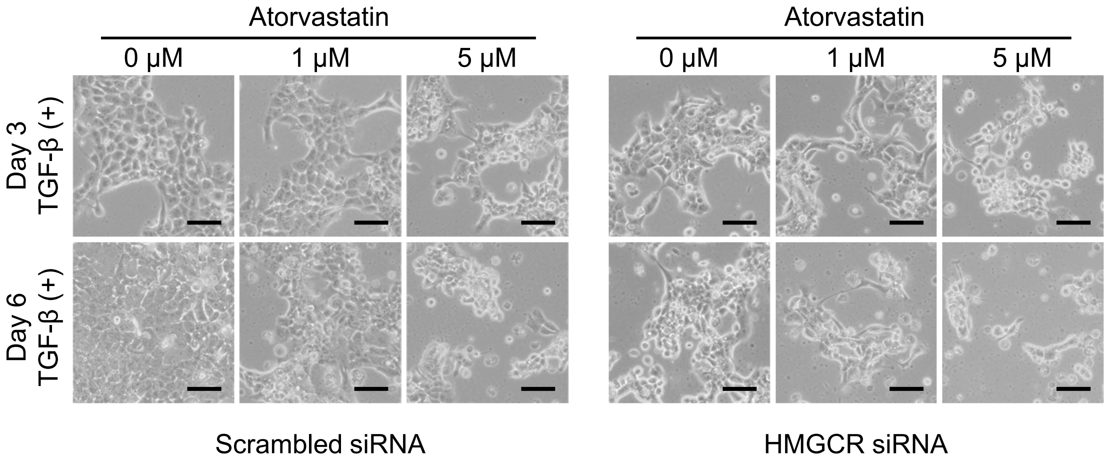

Fig. S7. Effect of *HMGCR* knockdown on cellular morphology of atorvastatin-pretreated TGF-β1 (+) group

Phase-contrast imaging at Day 3 (top) and Day 6 (bottom) on TGF-β-induced cells treated with 0–5 μM atorvastatin and *HMGCR* siRNA. Scrambled siRNA is used as negative control for RNAi experiment. Cell number decreases in an atorvastatin dose-dependent manner both in the scrambled controls (left panels) and *HMGCR* siRNA-treated cells (right panels). Additionally, the *HMGCR* knockdown diminishes the cells compared to that of scrambled controls. Scale bar =100 μm.

Table S1. Statistical analysis on cell number of NCI-H322M cells treated with atorvastatin and *HMGCR* siRNA

Comparison of experimental groups				p value
**Day 3**				
	Scrambled, 0 μM Atorvastatin	vs.	Scrambled, 1 μM Atorvastatin	<0.001
		vs.	Scrambled, 5 μM Atorvastatin	<0.001
		vs.	*HMGCR* siRNA, 0 μM Atorvastatin	<0.001
	Scrambled, 1 μM Atorvastatin	vs.	Scrambled, 5 μM Atorvastatin	<0.001
		vs.	*HMGCR* siRNA, 1 μM Atorvastatin	<0.001
	Scrambled, 5 μM Atorvastatin	vs.	*HMGCR* siRNA, 5 μM Atorvastatin	0.5367
	HMGCR siRNA, 0 μM Atorvastatin	vs.	*HMGCR* siRNA, 1 μM Atorvastatin	<0.01
		vs.	*HMGCR* siRNA, 5 μM Atorvastatin	<0.001
	HMGCR siRNA, 1 μM Atorvastatin	vs.	*HMGCR* siRNA, 5 μM Atorvastatin	1.0000
**Day 6**				
	Scrambled, 0 μM Atorvastatin	vs.	Scrambled, 1 μM Atorvastatin	<0.001
		vs.	Scrambled, 5 μM Atorvastatin	<0.001
		vs.	*HMGCR* siRNA, 0 μM Atorvastatin	<0.001
	Scrambled, 1 μM Atorvastatin	vs.	Scrambled, 5 μM Atorvastatin	<0.001
		vs.	*HMGCR* siRNA, 1 μM Atorvastatin	<0.001
	Scrambled, 5 μM Atorvastatin	vs.	*HMGCR* siRNA, 5 μM Atorvastatin	<0.001
	HMGCR siRNA, 0 μM Atorvastatin	vs.	*HMGCR* siRNA, 1 μM Atorvastatin	<0.001
		vs.	*HMGCR* siRNA, 5 μM Atorvastatin	<0.001
	HMGCR siRNA, 1 μM Atorvastatin	vs.	*HMGCR* siRNA, 5 μM Atorvastatin	0.0511

Table S2. Forward and reverse primer sequences for real time RT-PCR

Gene	Primer sequence	Product size (bp)	Gene accession number
E-cadherin	5′- GTCATCCAACGGGAATGCA -3′<br>5′- TGATCGGTTACCGTGATCAAAA -3′	60	NM_004360
N-cadherin	5′- ACCAGGTTTGGAATGGGACA -3′<br>5′- ACATGTTGGGTGAAGGGGTG -3′	156	NM_001792
Vimentin	5′- TCTACGAGGAGGAGATGCGG -3′<br>5′- GGTCAAGACGTGCCAGAGAC -3′	213	NM_003380
c-Myc	5′- TACAACACCCGAGCAAGGAC -3′<br>5′- TCGTCGCAGTAGAAATACGG -3′	250	NM_002467
HMGCR	5′- AGGAACCTGAAATTGAACTT -3′<br>5′- TAACTGTCGGCGAATAGATA -3′	200	NM_000859
18S rRNA	5′- AAACGGCTACCACATCCAAG -3′<br>5′- CCTCCAATGGATCCTCGTTA -3′	155	NR_003286
